# A Multi-Parameter Decoupling Method with a Lamb Wave Sensor for Improving the Selectivity of Label-Free Liquid Detection ^†^

**DOI:** 10.3390/s120810369

**Published:** 2012-07-31

**Authors:** Lianqun Zhou, Yihui Wu, Ming Xuan, Jean-François Manceau, François Bastien

**Affiliations:** 1 Suzhou Institute of Biomedical Engineering and Technology, Chinese Academy of Sciences, Suzhou 215163, China; E-Mails: zhoulq@sibet.ac.cn (L.Z.); xuanm@ciomp.ac.cn (M.X.); 2 State Key Laboratory of Applied Optics, Changchun Institute of Optics, Fine Mechanics and Physics, Chinese Academy of Sciences, Changchun 130033, China; 3 Institute FEMTO-ST, CNRS, Université de Franche-Comté, Besançon 25044, France; E-Mails: jfmanceau@femto-st.fr (J.-F.M.); fbastien@femto-st.fr (F.B.)

**Keywords:** Lamb wave sensor, density, sound velocity, viscosity, determination of multi-parameters

## Abstract

In this paper, a liquid multi-parameter decoupling method with only one Lamb wave sensor is presented. In a Lamb wave sensor, antisymmetric modes (A_01_ mode for low frequency, A_03_ mode for high frequency) and symmetric modes (S_0_ mode) are used to detect multiple parameters of a liquid, such as its density, sound velocity, and viscosity. We found they can play very different roles in the detections. For example, the A_01_ mode is very sensitive to the liquid's density but the A_03_ mode is sensitive to the sound velocity. Here, the A_0_ mode is used to identify the density of the detected liquid and with this density value we obtained the viscosity by the amplitude shifts of the S_0_ mode. This could be a way to distinguish an unknown liquid with high sensitivity or to solve the problem of selectivity of label-free detection on biosensors.

## 1. Introduction

Acoustic sensors have been widely used in chemical/biological fields with the label-free detection method to detect the mass changes on the sensor surface [1–17]. However, the selectivity of this method is often poor due to the absorption of non-target molecules, which are difficult to distinguish using only one-parameter sensors [18,19]. This has been a common problem of all kinds of label-free biosensors. The detection of multiple parameters using a multi-mode acoustic sensor significantly improves the label-free detection method. Acoustic waves which travel in a medium can have multiple modes and this character has been successfully used in a combined detection of density and viscosity for high viscosity solutions [20]. However, for low viscosity solutions like aqueous electrolytic solutions or bio liquids, it is still a challenge [9,20–24].

The micro Lamb wave sensor is a powerful tool for liquid detection because it is easier to get multi-mode vibration, and it performs with high sensitivity and low attenuation [25–29]. The two well-known basic modes, the antisymmetric mode (A_0_ mode) and the symmetric mode (S_0_ mode), have already been used for solving the problem of temperature compensation on a chip by the authors of [26,30]. The A_0_ mode has been used to measure the concentration of bio/chemical liquids, such as the concentration of methanol for direct methanol cell applications [18]. However, it shows uncertain frequency shifts direction with the concentration changes of the liquid [18,31]. In fact, the parameters of a liquid, like the density, acoustic sound velocity and viscosity, will work together for a mode. So it's hard to decouple them with only one mode.

In this work, both the antisymmetric mode and the symmetric modes are used to decouple the liquid physical parameters. The characters of A_03_ mode, with a wavelength of about one-third of A_01_ mode (low frequency of A_0_ mode), are investigated with the liquid loading for the first time. In order to discuss the response of Lamb wave sensor to the liquid loading, two types of experiments were set up. The first experiment was used to measure the frequency shifts with the same type of solutions with different concentrations loaded on the sensors' surface, such as the solutions of NaCl. It shows that the characters of the modes are very different. Although both A_01_ and A_03_ modes belong to A_0_ mode, their frequency shifts showed an opposite behavior with the increase of the concentration of the loading solutions. The second experiment was used to measure the frequency changes with different types of solutions and different concentrations, such as solutions of KCl, NaBr and KBr. Although the tested objects are two different kinds of solutions with different concentrations, the frequency of A_01_ mode shows almost has no movement and A_03_ mode frequency shows a great difference. The frequency shifts of S_0_ mode for these solutions are not apparent. These will make it possible to be only sensitive to the liquid viscosity except for the environmental temperature. This essay attempts to decouple the functions of A_01_, A_03_ and S_0_ modes to obtain the basic physical parameters (density and sound velocity, viscosity) with different liquid loading responses. From the values which have been reported from literature [32] we can also determine the types of the solution. This work provides a selective, sensitive method for the measurement of physical parameters to an unknown solution. It can be useful for label-free biosensors.

## 2. Working Principle

Multi-mode wave, such as A_01_ mode, S_0_ mode and their harmonic ones (A_03_ mode, for example) can be excited in Lamb wave sensors and detected directly by using a pair of inter-digital transducers (IDTs) [33] located on the surface of the piezoelectric layer [34,35]. In liquid sensing, the liquid and IDT will be located on the two opposite sides of the membrane. For the A_01_ mode and the A_03_ mode, when the Lamb wave phase velocity is less than the liquid sound velocity, there are evanescent waves produced around the membrane-liquid interface. The phase velocity of the Lamb waves within the evanescent penetration field will be the same with the phase velocity inside the membrane. Taking account of the bending stiffness and the in-plane tension of the plate (B_i_) for the A_01_ mode and the A_03_ mode, the phase velocity will be [31,36]:

(1) $$c_{P_{i}}=\sqrt{B_{i}/(M+m_{Li})}$$

where *i* denotes 1 or 3 for the A_0i_ mode, *B*_i_ reflects the influences of the bending stiffness and the in-plane tension of the plate for the A_0i_ mode, *M* is the mass per unit area of the plate, the effective mass *m_Li_* in the so-called evanescent penetration depth *δ_Ei_* equals:

(2) $$m_{Li}=\rho_{L}\delta_{Ei}$$

In which, *ρ_L_* is the detected liquid density. The evanescent penetration depth *δ_Ei_* obeys the following equation [37–39]:

(3) $$\delta_{Ei}=\lambda_{i}/(2\pi \sqrt{1-{\left(c_{Pi}/c_{L}\right)}^{2}})$$

where *λ_i_* and *c_Pi_* denote the Lamb wave wavelength and phase velocity respectively in each mode. *c_L_* is the bulk acoustic velocity of the detected liquid. *c_Pi_* is determined by the frequency (*f_i_*) via *c_Pi_* = *f_i_ λ_i_*.

By substituting the Equations (2) and (3) into the Equation (1), we will get the following formula:

(4) $$\rho_{L}\lambda_{i}/\left(2\pi \sqrt{1-{\left(c_{Pi}/c_{L}\right)}^{2}}\right)=B_{i}/c_{Pi}^{2}-M$$

In this formula, the constants *B_i_, λ_i_*, and *M* are independent of the liquid type; *λ_i_* is determined by the structure of the device. With the implicit Equation (4), the density and sound velocity of the liquid on the sensor can be decoupled when the frequency response of the A_01_ mode and the A_03_ mode are obtained by the experiments.

In the measurement, the relative frequency shifts (Δ*f/f*) will be used frequently. By taking the term *c_Pi_* = *f_i_ λ_i_* into Equation (1) and deriving of the equation, the value of Δ*f/f*) for the A_01_ mode and the A_03_ mode will be:

(5) $$\Delta f_{i}/f_{i}=-\Delta (\rho_{L}\delta_{Ei})/2M=-\left(\delta_{Ei}\Delta \rho_{L}+\rho_{L}\Delta \delta_{Ei}\right)/2M$$

Obviously, the density and the sound velocity are the two factors affecting the relative frequency shifts. When the phase velocity is far less than the liquid sound velocity (*c_P_* « *c_L_*), such as the A_01_ mode, the values of *δ_E_* and Δ*δ_E_* approach *λ*/2*π* and 0, respectively. Then, in this situation the variations of the liquid sound velocity will have an almost negligible influence on the relative frequency shift. When the phase velocity is close to the liquid sound velocity, such as the A_03_ mode, the value 1 – (*c_P_/c_L_*)^2^ approaches 0. The influence of the terms *δ_E_* and *c_L_* cannot be neglected any more. The liquid density and sound velocity will simultaneously affect the frequency shifts. The value of Δ*f/f* can be positive or negative which depends on the sum of the value of *ρ_L_*Δ*δ_E_* and the value of *δ_E_*Δ*ρ_L_*. Anyway, if we combine these two cases (A_01_ mode, A_03_ mode) simultaneously, we can get the values *ρ_L_* and *c_L_* with high sensitivity.

For the S_0_ mode, when the wavelength of the Lamb mode is larger than the thickness of the plate, the phase velocity (*c_p_*) for the principal symmetric mode can be simplified as [40]:

(6) $$c_{p}=c_{t}{\left[4\left(1-\frac{c_{t}^{2}}{c_{d}^{2}}\right)\right]}^{\frac{1}{2}}\left[1+\frac{I}{2}\frac{\rho_{L}c_{L}^{2}}{\rho_{m}c_{t}^{2}}\frac{\omega d}{2c_{L}}\left(\frac{1}{4\left(1-c_{t}^{2}/c_{d}^{2}\right)}-\frac{c_{t}^{2}}{c_{d}^{2}}\right)\right]$$

where *c_d_* and *c_t_* are the velocities of longitudinal (dilatational) and transverse (shear) waves of the membrane, *c_L_* is the liquid sound velocity, *ρ_m_* and *ρ_L_* are the density of the solid membrane and liquid respectively, *d* is the membrane thickness, *I* denotes the square root of −1.

Evidently, the effect of liquid on the propagation of the S_0_ mode does not change the real part of the phase velocity, but adds a very small attenuation of the amplitude [40]. This mode is suitable for the detection of attenuation. The amplitude (*A_L_*) response of an unknown solution can be expressed by:

(7) AL=A0e−αxXL

where the attenuation coefficient *α_XL_* is proportional to (*ρ_L_η_L_*)^0.5^, with *η_L_* being the viscosity of the liquid. Similarly, the amplitude response (*A_W_*) of water is given by:

(8) AW=A0e−αxXW

In engineering, the insertion loss (dB) is widely used. The values of *A_L_* and *A_W_* can be transformed into values in dB scale which are denoted by *A_LdB_* and *A_WdB_*, respectively. Therefore, the attenuation difference (Δ*A_dB_*) between an unknown solution and water can be expressed in dB scale as follows:

(9) $$\Delta A_{dB}=A_{\text{LdB}}-A_{\text{WdB}}=\gamma_{1}{\left(\rho_{L}\eta_{L}\right)}^{1/2}+\gamma_{2}$$

in which both of the slope *γ*_1_ and *γ*_2_ are constant, and the constant *γ*_2_ is determined by the reference liquid (water). As the density and the sound velocity of the solution can be estimated by both the frequency shifts of the A_01_ mode and the A_03_ mode, the multi-parameters (*ρ_L_, c_L_, η_L_*) of the solution can be decoupled simultaneously with these three modes. According to these principles, a series of experiments were set up to investigate the responses of the multi modes of Lamb wave to the loading solutions.

## 3. Experiments for Liquid Detection

### 3.1. Experimental Setup

**Figure 1.**
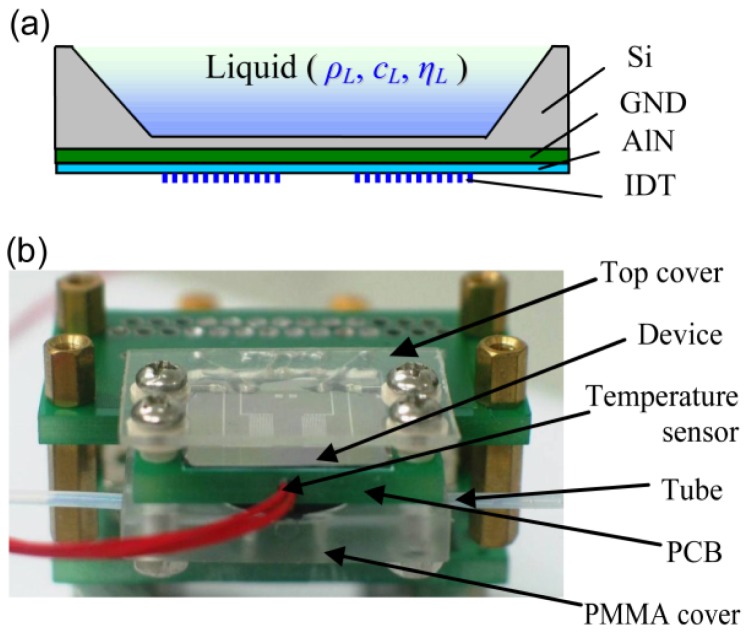
The system for liquid detection, (**a**) Schematic diagram of the micro Lamb wave sensor interaction with liquid, *ρ_L_*: density, *c_L_*: sound velocity, *η_L_*: viscosity; (**b**) Micro Lamb wave sensor packaged with printed circuit board (PCB).

The micro Lamb wave device in Figure 1(a) contains a silicon membrane (length 7.8 mm, thickness ∼12 μm) with a ground layer (Ti/Mo, GND, ∼0.2 μm) and a piezoelectric layer (aluminum nitride, AlN, ∼1.8 μm). Lamb waves are excited and detected directly using bidirectional inter-digital transducers (IDTs) [33] located on the surface of the AlN layer. There are six pairs of fingers on bidirectional IDTs in each exciting and detecting transducer. The period of bidirectional IDT is about 400 μm. As waves are partly reflected at the end of length-limited membrane (∼7.8 mm), the device has strong signal without reflectors on both ends of the membrane. With the layers described as in [34], the mass per unit area of the membrane (*M*) is about 0.0355 kg/m^2^.

The micro Lamb wave device is packaged directly with the printed circuit board (PCB), as shown in Figure 1(b). The network analyzer (Agilent 4395A), connected with the PCB, is used to excite and receive the acoustic signals. The device is protected with one cover on top of the system. The tube is used to pass the liquid into/out of the chamber, which is sealed up with the polymethyl methacrylate (PMMA) cover under the PCB.

**Figure 2.**
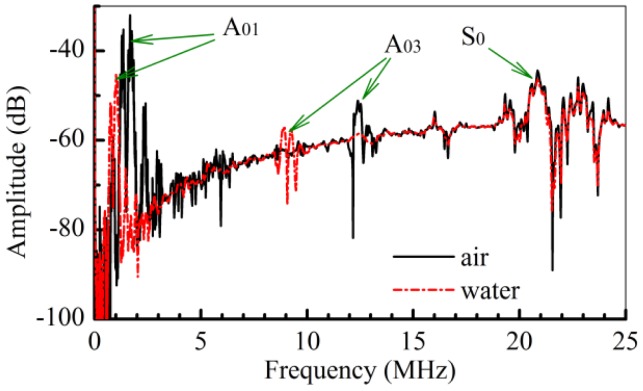
The responses of the A_01_ mode, the A_03_ mode, and the S_0_ mode to the air and the water loading on the Lamb wave sensor.

When the device is loaded with air or water, multi-modes can be excited and detected effectively, including the A_01_ mode, the A_03_ mode, and the S_0_ mode (Figure 2). As the A_01_ and A_03_ modes are harmonic, the wavelength of the A_03_ mode is about one third of the wavelength of the A_01_ mode. According to the positions of the A_01_ mode, A_03_ mode and S_0_ mode in the dispersion curves [41,42], the resonant frequencies (*f*) of these three modes should have the following relation: *f_A01_* < *f_A03_* < *f_S0_*. The measured resonant frequency of the A_01_ and A_03_ modes for water loading are 0.987 and 9.233 MHz, respectively. With the frequency (*f*) and the wavelength (*λ*), the corresponding phase velocities (*fλ*) of these two modes are 385 m/s and 1,200 m/s respectively. Although the phase velocity of the A_01_ mode is far less than the sound velocity of water (1,485.5 m/s), it is close to the sound velocity of water for the A_03_ mode. With the same water loading for the S_0_ mode, the measured central resonant frequency is about 20.86 MHz. The corresponding phase velocity of the S_0_ mode is about 8,135.4 m/s. Further experiments were conducted to investigate the response of multi-modes to the different solutions.

### 3.2. Experiments for One Species Solutions (NaCl Solutions) with Different Concentrations

**Figure 3.**
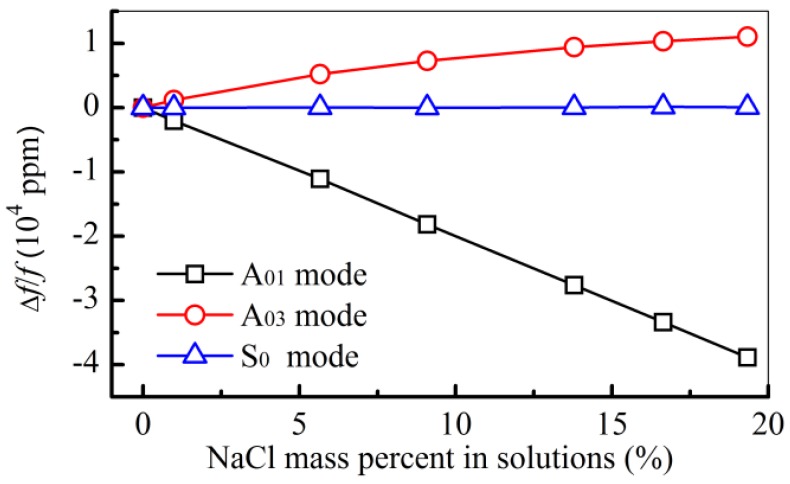
Relative frequency shifts (Δ*f/f*) for the A_01_ mode, the A_03_ mode, and the S_0_ mode in the measurements of the NaCl solutions.

The first experiment was done to measure known solutions with different concentrations (Figure 3), such as NaCl solutions. When water is designated as the reference liquid, the relative frequency shifts (Δ*f/f* = (*f*_solution_ − *f*_water_)/*f*_water_) with concentration are different for these three modes (A_01_ mode, A_03_ mode, and S_0_ mode), where *f*_solution_ and *f*_water_ are the measured frequencies for the solution and the water, respectively. The frequency of the A_01_ mode decreases with the concentrations and the A_03_ mode behaves oppositely. In the case of the A_03_ mode of NaCl solutions measured in Figure 3, the absolute value *ρ*Δ*δ_E_* is bigger than the absolute value *δ_E_*Δ*ρ* (Table 1). This causes the positive frequency shifts of A_03_ mode in NaCl solutions measurements. In any case, when the frequency shift is positive, this means the Lamb wave phase velocity is close to the liquid sound velocity. In this case, this phenomenon can be used to decouple the liquid sound velocity.

Compared with the frequency shifts measured with the A_01_ mode and A_03_ mode, the frequency of the S_0_ mode shows negligible shift with different concentrations (Figure 3).

### 3.3. Experiments for Three Different Unknown Species Solutions

The second experiment was done to measure the modes' responses to three different species of aqueous electrolytic solutions with different concentrations (Table 2). We will check the possibility of the method to decouple the density and the acoustic sound velocity of these solutions with the measurements of A_01_ mode and A_03_ mode. Three different species of aqueous electrolytic solutions are prepared, they are listed as solution A, solution B, and solution C respectively, as it is shown in Table 2. From No. 1 to No. 6 solutions, they are the solution A. No. 7 and No. 8 solutions are the solution B. No. 9 and No. 10 are solution C. In each kind of solution, the concentration increases with the serial number. The S_0_ mode is still insensitive to the solution changes compared with the measurement in water, and its values are not listed here.

In the case of solution A, the relative frequency shifts Δ*f/f* of A_01_ mode and A_03_ mode have similar properties with the solution of NaCl measured in Figure 3. But for the solution B and solution C, the relative frequency shifts of A_03_ mode is negative. As it is indicated in Equation (5), the absolute value *ρ*Δ*δ_E_* of solution B and solution C measured in Table 2 should be smaller than the absolute value *δ_E_*Δ*ρ*.

Even for different kinds of solutions, such as the No. 6, No. 8, and No. 10 solutions, the values of Δ*f/f* of the A_01_ mode are almost the same, but the values of Δ*f/f* of the A_03_ mode are apparently different. This means only one antisymmetric mode measurement, such as A_01_ mode, can not determine the solution with high selectivity. In order to specify the liquid with high selectivity, it is better to decouple the liquid density and the liquid sound velocity with the measured relative frequency shifts of A_01_ mode and A_03_ mode. These will be analyzed in following section.

### 3.4. Viscosity Measurements with the S_0_ Mode

**Figure 4.**
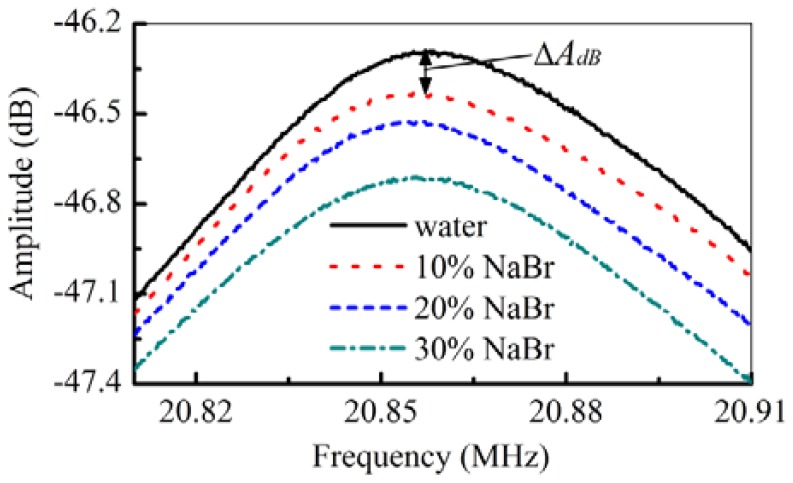
The responses of the S_0_ mode to different concentrations of NaBr solutions.

The responses of the S_0_ mode to the solutions of NaBr are similar to the ones of NaCl (Figure 3), the central frequency does not show apparent shifts due to their changing concentrations. However, the changes of the amplitude are related to the liquid's concentrations (Figure 4). It is because the frequency is mainly affected by the liquid density and sound velocity, which has no effects on the real part of the phase velocity of S_0_ mode (Equation (6)) [40]. The amplitude of the S_0_ mode in NaBr solution (Figure 4) shows that energy losses increase with the concentration, which is caused by the density and the viscosity of the liquid (Equation (7)). This can be used to decouple the density and the viscosity of the solution.

## 4. Results and Discussions

### 4.1. To Decouple the Density and the Acoustic Sound Velocity with the A_01_ Mode and A_03_ Mode

In order to specify the liquid with high selectivity, we attempted to decouple the liquid density and the liquid sound velocity with the measured frequency shifts of A_01_ mode and A_03_ mode. Based on the Equation (4), two steps are undertaken in order to decouple the density and the sound velocity of a liquid as follows:

1. Determine the constant *B_i_* by measuring the frequency response of the reference liquid. Here, the water works as the reference liquid, the values of *B*_1_ and *B*_3_ are 14,767.3 N/m and 101,642.8 N/m respectively.
2. Get the physical parameters (*ρ_L_, c_L_*) for a unknown liquid by measuring *c_Pi_*. All other parameters (*B_i_, λ_i_, M*) in Equation (1) have already been calculated or measured.

**Figure 5.**
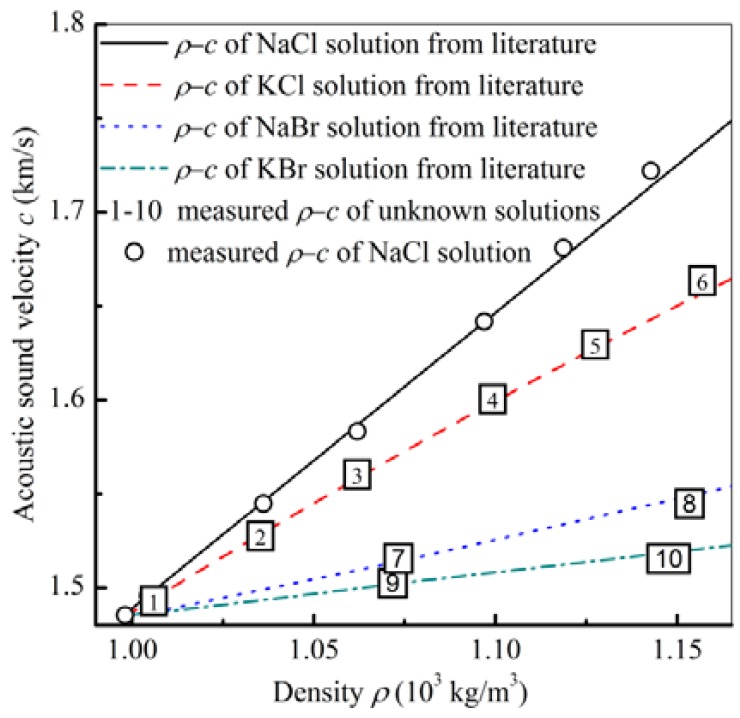
The decoupled density and sound velocity of NaCl solutions and other solutions using measurements in A_01_ mode and A_03_ mode.

The first decoupled result using Equation (1) is the NaCl solution with different concentrations, as measured in Figure 3. Comparing the measured density and sound velocity with the values from the literature [32], the two results are close, as shown in Figure 5.

Based on the same process, the density and the sound velocity for other three different species solutions with different concentrations were also decoupled and are shown in Figure 5. Compared with the values from the literature [32], No. 1–No. 6 solutions are KCl solutions, No. 7 and No. 8 solutions are NaBr solutions, and No. 9 and No. 10 solutions are KBr solutions. Aside from decoupled density and sound velocity, the species of the solutions can be identified by comparing the measured values with the already known values [32]. After these density and sound velocity being decoupled using A_01_ mode and A_03_ mode, the liquid are determined with higher selectivity compared with only A_01_ mode measurement.

For Nos. 6, 8, and 10 solutions, the decoupled densities (Figure 5) are almost the same because the relative frequency shifts are very close (Table 2). For these solutions with adjacent density, sound velocities become the main factors affecting the frequency shifts in the A_03_ mode (Table 2). With the relations of the absolute frequency shifts: Δ*f/f*_No.6_ > Δ*f/f*_No.8_ > Δ*f/f*_No.10_ (Table 2), the decoupled sound velocity of these three solutions have the following relation: c_No.6_ > c_No.8_ > c_No.10_ (Figure 5).

### 4.2. The Linear Response of (Density × Viscosity)^0.5^ Changing with the Item of Amplitude Shifts (ΔA_dB_) in S_0_ Mode

**Figure 6.**
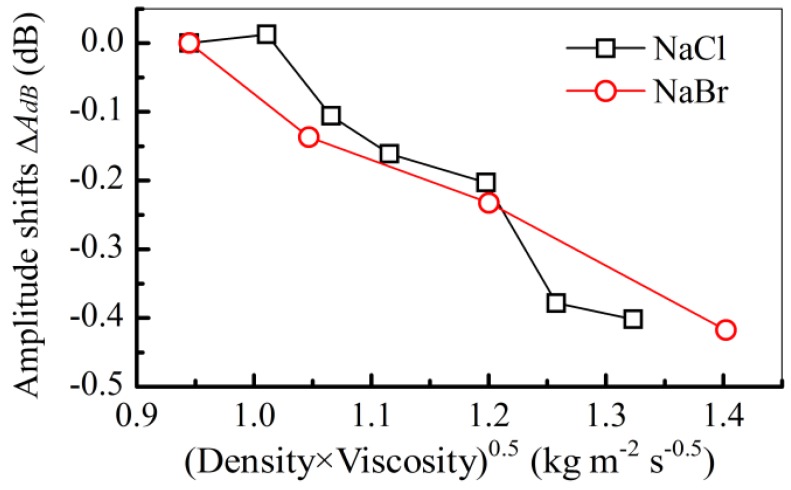
Amplitude shifts *versus* (density × viscosity)^0.5^ for the NaCl and NaBr solutions in the S_0_ mode.

The item (density × viscosity)^0.5^ changes almost linearly with the item of amplitude shifts (Δ*A*_dB_), and the linear fitting coefficient is about 1.02 ± 0.09 (dB/(kg·m^−2^·s^−0.5^)), such as the NaBr and NaCl solutions (Figure 6). Beside the density and the viscosity, other parameters, such as the liquid's conductivity, affect the amplitude shifts. This is maybe the reason why the high linearity of (density × viscosity)^0.5^ doesn't change with the amplitude shift. However, by analyzing the amplitude shifts (Δ*A*_dB_) of the S_0_ mode, the viscosity of the solution can be derived. With the determined density by measuring the frequency response of the A_01_ and A_03_ modes, the viscosity will be determined by checking the amplitude response in the S_0_ mode.

## 5. Conclusions

In this study, for the first time to our knowledge, the density, sound velocity, and viscosity of the liquid are obtained simultaneously based on the measurements of the same solution sample volume with a Lamb wave sensor. When the frequency shifts of the A_01_ and A_03_ modes are combined, the density and sound velocity are decoupled. This happens because the phase velocity of the A_01_ mode is far from the sound velocity of the liquid and the phase velocity of the A_03_ mode is close to the sound velocity of the liquid. Viscosity is obtained by measuring the amplitude of the S_0_ mode, which is especially suitable for Newtonian fluids. Effects of the aqueous electrolytic solutions' conductivity on these three modes are not clearly observed. The term (density × viscosity)^0.5^ doesn't show high linearity as a function of amplitude shifts.

However, with this multi-parameter detection method, unknown solutions such as aqueous electrolytic solutions can be distinguished successfully and with high selectivity. Results clearly indicate that the multi-modes of a micro Lamb wave sensor are promising in the investigation of molecular thermodynamics, adiabatic compressibility, and molecular label free detection, among others.

## Figures and Tables

**Table 1.** Based on the measured frequency shifts of NaCl solutions measured in Figure 3, comparison of the value *ρ*Δ*δ_E_* and the value *δ_E_*Δ*ρ* for the A_03_ mode.

**Concentration (%)**	**Density (10^3^ kg/m^3^)**	*c_P_* **(m/s)**	*δ_E_* **(μm)**	*δ_E_*Δ*ρ*	*ρ*Δ*δ_E_*
0	0.9981	1,200.30	35.12	0	0
0.99	1.0052	1,201.71	34.70	0.25	−0.42
5.67	1.0389	1,206.55	32.81	1.34	−2.39
9.09	1.0640	1,209.03	31.58	2.08	−3.77
13.79	1.0995	1,211.59	30.01	3.04	−5.62
16.64	1.1212	1,212.69	29.13	3.59	−6.71
19.33	1.1425	1,213.53	28.40	4.10	−7.67

**Table 2.** Relative frequency shifts (Δf/f) for the A_01_ mode and the A_03_ mode in the measurements of three kinds of unknown solutions. In each kind solution, the concentration increases with the serial number.

	**Solution A**						**Solution B**		**Solution C**	

**No. of solutions**	1	2	3	4	5	6	7	8	9	10
Concentration (%)	0.99	6.05	9.97	16.03	19.96	23.95	9.35	18.07	8.98	17.32
Δ*f/f* (A_01_ mode) (−1 × 10^4^ ppm)	0.236	1.09	1.846	2.886	3.654	4.434	2.243	4.474	2.216	4.356
Δ*f/f* (A_03_ mode) (10^4^ ppm)	0.027	0.216	0.357	0.373	0.325	0.287	−0.649	−1.422	−0.876	−1.848
